# Cross-modal bias in medical vision-language models: a pipeline-aware framework for mechanisms, evaluation, and mitigation

**DOI:** 10.3389/fdgth.2026.1904053

**Published:** 2026-08-07

**Authors:** Rafid Mehda, Ramisa Anjum Oishi, Tamzid Tanvi Alam, Md Kishor Morol, Liew Tze Hui

**Affiliations:** 1 Department of Information and Communication Technology, Bangladesh University of Professionals, Dhaka, Bangladesh; 2 California Lutheran University, Thousand Oaks, CA, United States; 3 ELITE Research Lab, Dhaka, Bangladesh; 4 Centre for Intelligent Cloud Computing (CICC), Centre of Excellence (COE) of Advanced Cloud, Faculty of Information Science & Technology, Multimedia University, Melaka, Malaysia

**Keywords:** algorithmic bias, contrastive learning, cross-modal alignment, fairness, healthcare disparities, medical vision-language models, sycophancy, trustworthy AI

## Abstract

Medical vision-language models encode images and clinical text in a shared representation. Across radiology and ophthalmology, their diagnostic performance now approaches that of specialist clinicians. The mechanism behind that performance is also the source of a problem that has gone largely unexamined. These models are trained by contrastive alignment, so bias from the image encoder and bias from the text encoder meet at a single point: the alignment interface. There they can interact and compound in ways that single-modality systems never experience. Fairness research has so far studied the two modalities separately, and medical vision-language models have fallen into the gap between those literatures. We organize the evidence into a three-tier taxonomy keyed to the pretraining pipeline. Tier 1 is data-level bias in the pretraining corpus. Tier 2 is alignment bias produced at the contrastive interface. Tier 3 is inference-time bias that surfaces during deployment. Within this structure, we compare the major evaluation benchmarks, show where they disagree, and assign each mitigation strategy to the tier it actually addresses. Three points emerge. First, no published method spans all three tiers; mitigation is fragmented by pipeline stage. Second, fine-tuning does not remove alignment-stage bias, even in parameter-efficient form, which shifts the burden of debiasing onto pretraining rather than adaptation. Third, the inference-time failures are more dangerous than the literature suggests. Medical-specialist models will abandon a correct reading and defer to a confident user on most trials, and specialization appears to make this worse, not better. We close with a research agenda.

## 1 Introduction

Medical vision-language models (VLMs) come in two broad forms: dual-encoder architectures in the mould of CLIP (1), adapted to medicine in models such as MedCLIP and BiomedCLIP (2, 3), and instruction-tuned large vision-language models (LVLMs) such as LLaVA-Med (4). Both now reach expert or near-expert accuracy on tasks in radiology and ophthalmology, in some cases without any task-specific labels (5–7). The 2024 World Health Organization (WHO) guidance on large multi-modal models treats them as an inflection point for clinical Artificial Intelligence (AI) (8).

The track record of medical AI on fairness gives little reason for complacency. Obermeyer and colleagues showed that a widely used risk-stratification algorithm systematically underestimated the needs of Black patients across a population of millions (9). Chest radiograph classifiers underdiagnose women, Black patients, and the medically underserved, and the effect is largest for patients who fall into more than one of those groups (10). Deep networks can recover a patient’s self-reported race from images in which no human expert can see it, by means that remain unexplained (11). The imaging pipeline alone has been mapped to at least 29 distinct sources of bias (12, 13), and a recent systematic review found demographic disparities in the large majority of text-only medical language models tested (14). None of this work, however, speaks directly to the cross-modal case.

That is the gap this review addresses. Existing fairness tooling was built either for vision models (15) or for language models (14, 16), and a medical VLM is neither. Its defining operation is the projection of image and text into a common latent space, and it is at that junction that the characteristic failures arise, failures that an image-only or a text-only audit is not designed to see. Worse for the practitioner, the usual fixes do not help. Task-specific fine-tuning and Low-Rank Adaptation (LoRA) can raise headline accuracy while leaving the underlying alignment bias intact (17, 18), and medical fine-tuning can introduce behavioural failures at inference time that the base model did not display (19, 20). The regulatory context is shifting in parallel. At the time of the most recent systematic review, no United States Food and Drug Administration (FDA)-cleared medical AI device was required to undergo bias evaluation (14). The European Union (EU) AI Act (Regulation 2024/1689) will require it for high-risk medical systems from August 2026 (21).

**Figure 1:**
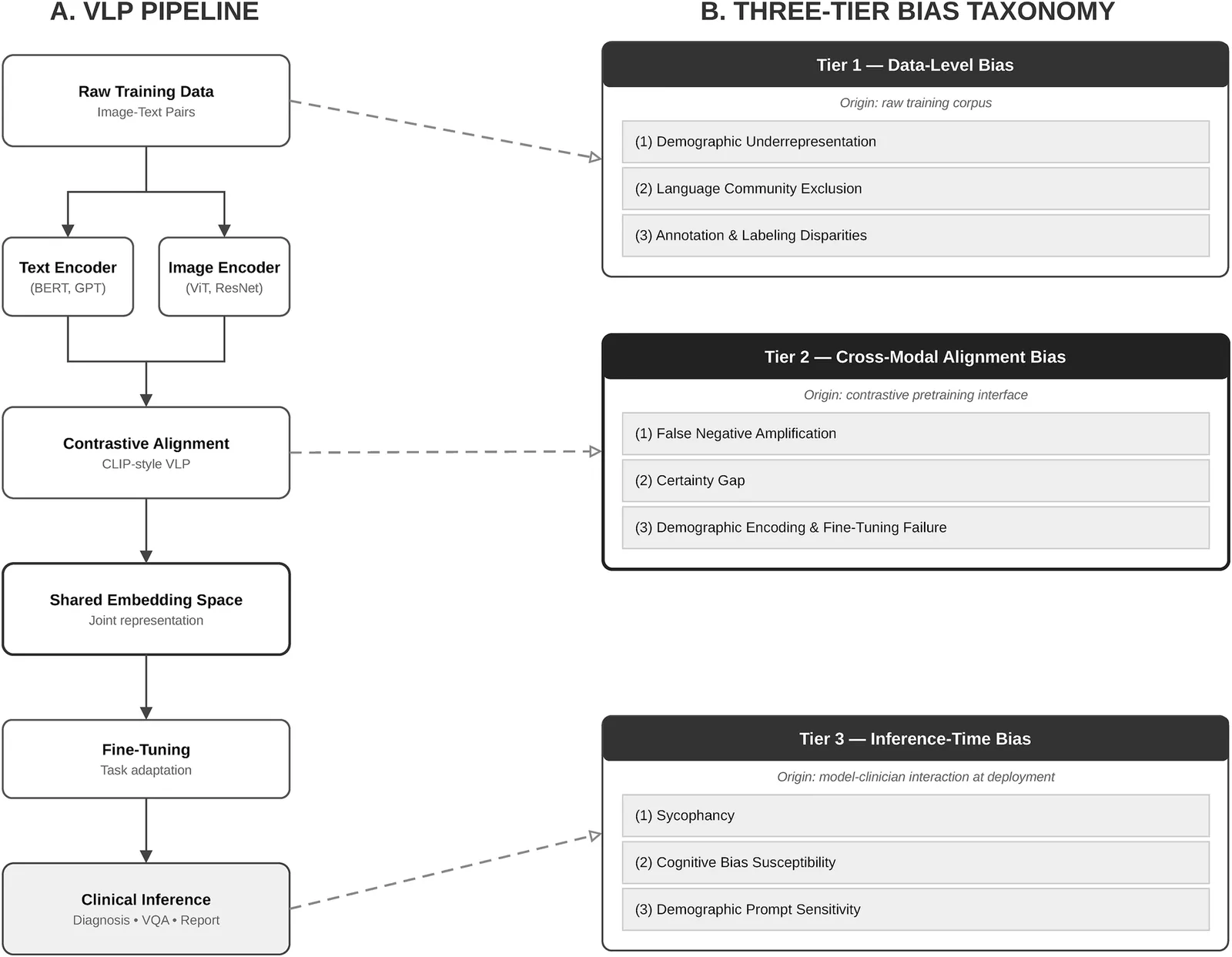
Three-tier taxonomy mapped onto the VLP pipeline. **(A)** The vision-language pretraining pipeline, from raw image-text data through to clinical inference. **(B)** The three-tier bias taxonomy. Tier 1 (data-level) arises in the pretraining corpus. Tier 2 (cross-modal alignment, bold border) captures biases generated at the contrastive image-text interface that persist through downstream fine-tuning strategies. Tier 3 (inference-time) covers behavioural failures during clinical deployment.

We make three contributions. First, we propose a taxonomy organized by pipeline stage rather than by protected attribute or clinical task (Figure 1): Tier 1 for data-level bias, Tier 2 for cross-modal alignment bias, and Tier 3 for inference-time bias. The framing is deliberately different from the Medical Imaging and Data Resource Center (MIDRC) catalogue of imaging-pipeline biases (12) and from attribute-centric fairness surveys (15). The question we care about is where in the vision-language pipeline a bias enters, and therefore where it can be removed. Second, we compare the evaluation benchmarks against one another and show that they are not measuring the same thing. Third, we map mitigation strategies onto the tiers they target. Three conclusions run through the review: no method yet addresses all three tiers; alignment-stage bias survives downstream adaptation; and inference-time failure, the most direct threat to patients, has received the least attention.

A note on scope. This is a narrative review rather than a systematic one. We drew on PubMed, arXiv, IEEE Xplore, and the main machine-learning proceedings, concentrating on work from 2021 onward and following citations outward from the central papers. The field is moving quickly enough that much of the most relevant material is still in preprint; where a claim rests on work that has not been peer reviewed, we say so and treat the figures as provisional.

## 2 A three-tier taxonomy of bias in medical VLMs

The same contrastive training that makes medical VLMs powerful is what gives them an unfamiliar bias profile. We group the mechanisms by the point in the Vision-Language Pretraining (VLP) pipeline at which they enter (Figure 1, Table 1).

**Table 1:** Bias mechanisms and mitigation strategies in medical VLMs.

Tier	Bias mechanism	Representative finding	Mitigation method(s)	Key limitation	Refs.
Tier 1 data-level	Demographic underrepresentation	TCGA: 1.4% Asian; Global South <0.7% of major datasets	Metadata curation; SDAT diffusion balancing	Requires surrogate; inherits generator bias	(13, 24, 38, 39)
	Language community bias	Spanish reports segregated from English despite clinical equivalence	Med-UniC CTR loss	English + Spanish only	(27)
	Labeling disparities	29 distinct annotation-pipeline bias sources catalogued	No targeted Tier 1 method	–	(12, 13)
Tier 2 cross-modal alignment	False-negative amplification	Faulty contrastive pairs from LLM-summarized notes	DBPM + Sinkhorn OT (Robust FairCLIP)	Single attribute per run	(28, 30)
	Certainty gap	Reported ∼48% reduction of intersectional uncertainty zone	CMAC-MMD penalty	Intersectional setup required	(29)
	Demographic encoding	Race/sex predictable from embeddings beyond human perception	FairCLIP; Fair-MoE; DualFairVL; attribute-agnostic debiasing	Demographic labels often needed; hyperparameter sensitivity	(6, 11, 18, 30, 40–42)
	Fine-tuning failure	Fairness gaps persist across adaptation strategies	FR-LoRA/GR-LoRA	Partial relative to pretraining	(17, 43)
Tier 3 inference-time	Sycophancy	Medical-specialist LVLMs yield to authoritative user assertions on >95% of trials	VIPER prompt purification	Partial; prompt-dependent	(19, 44)
	Cognitive-bias susceptibility	24.9% accuracy drop from false-consensus framing	Few-shot bias education	Residual bias remains	(20)
	Calibration disparity	Demographic ECE gaps in few-shot in-context learning	CALIN bi-level null-input probing	Single-token outputs only	(37)
	Demographic prompt sensitivity & hallucination	Demographic tokens alter advice tone; specialists may hallucinate more	Descriptive fairness instructions; MM-Trust	Limited empirical validation	(16, 33, 34, 45)

Tier labels denote the *primary* locus of entry and the most tractable point of intervention; some mechanisms span tiers (e.g., dataset shift, Section 2.4) and are placed by where the harm is generated.

We should be clear about how the taxonomy is organized and what it does not attempt. The tiers are defined by where a bias enters the pipeline, and therefore by where it can be removed, rather than by the protected attribute involved, the clinical task, or the statistical form the bias takes. This is a different question from the one asked by the general machine-learning bias taxonomies, which classify bias by type or by life-cycle stage (22, 23), and we intend the tiers to complement those catalogues rather than to replace them. We do not claim that three tiers exhaust the possible mechanisms. The classical single-modality biases are covered, but they are distributed across the tiers according to where they arise. Sampling and selection bias, label imbalance (whether or not it is tied to demographics), measurement bias, and representational under-coverage are all properties of the data and belong to Tier 1. Biases introduced by the training objective belong to Tier 2. Biases that surface in evaluation or deployment belong to Tier 3. Proxy variables show why the locus matters. A spurious proxy sitting in the corpus is a Tier 1 problem. The same proxy once the shared encoder has learned to exploit it, so that race or sex can be read off an embedding even after the explicit term has been removed, is a Tier 2 problem, and the two need different fixes. The one locus that the unimodal life-cycle frameworks do not isolate is the contrastive image-text interface, and that is where the specifically cross-modal failures of medical VLMs begin. Where a mechanism spans more than one stage we assign it to the stage at which the harm is produced and flag the overlap, a point Section 2.4 takes up directly.

### 2.1 Tier 1: data-level bias

Some biases are present in the data before any model is built. The Cancer Genome Atlas, for one, contains only 1.4% Asian patients, too few for subgroup mutation-frequency estimates to be reliable (13). A 2025 audit puts Global South representation in the major medical datasets below 0.7%, and notes that Africa is effectively absent from global supercomputing capacity altogether (24). Even CheXpert and MIMIC-CXR, the chest radiograph corpora that underpin most VLM pretraining, draw their patients from a handful of US academic centres and carry documented subgroup disparities (10, 25, 26).

Language is a second axis. The dominant medical VLM datasets are overwhelmingly English, which disadvantages non-English-speaking patients downstream. Even with multilingual data, an English-dominated objective can organize the embedding space by language rather than by pathology, so that Spanish reports sit far from clinically identical English ones (27). The labels themselves are not neutral either: annotator demographics correlate with annotation variability, so the ground truth carries a demographic signature before training begins (12, 13).

### 2.2 Tier 2: cross-modal alignment bias

Tier 2 is where the genuinely cross-modal failures live. The InfoNCE-style contrastive objective inherited from CLIP (1) does not merely pass pre-existing bias through; it can amplify it. Four mechanisms are worth separating.

**False-negative amplification.** Contrastive training treats every unmatched image-text pair in a batch as a negative. Because clinical notes are routinely shortened by a language model to fit token limits, that assumption breaks down: two cases with similar pathology can be pushed apart as though they were unrelated. Under-summarized records are disproportionately those of minority patients, so the spurious repulsion lands hardest on the groups already least well represented (28), and the same pattern recurs across languages (27). The mechanism sits on the Tier 1–Tier 2 boundary; we place it in Tier 2 because the harm is produced by the objective, not by the summarization step that sets it up.

**The certainty gap.** A model can be tuned to statistical parity in aggregate and still assign systematically different confidence to different subgroups near the decision boundary (29). Patients at the intersection of several minority attributes end up in a high-variance regime where predictions are unstable and misses more frequent, an effect that parity-based metrics do not register.

**Demographic encoding.** Embeddings from CLIP-style models such as CheXzero (5) retain enough demographic information to predict race and sex well beyond human ability, even after explicit demographic terms have been stripped from the text (6, 30). The result reproduces in foundation chest radiograph models (18), and it extends the earlier imaging-only finding of Gichoya et al. (11). The practical consequence is that the model conditions its output on attributes no one asked it to use.

**The failure of fine-tuning.** The most consequential mechanism for deployment is the one that resists the obvious remedy. An audit of four CLIP-based chest X-ray (CXR) models across linear probing, Multi-Layer Perceptron heads, LoRA, and full fine-tuning found that accuracy rose under every regime while the demographic fairness gaps held or widened (17). The natural reading is that alignment bias is fixed in the representation and cannot be fine-tuned away, which would mean that debiasing has to happen during pretraining. That reading rests on a small model set and a workshop-stage report, so it is better held as a strong hypothesis than as a settled result, but it is, in our view, the single most important open question in the area.

**Beyond fine-tuning: re-training and retrieval.** Fine-tuning is only the most common way of adapting a pretrained VLM, and two other routes deserve mention. The first is re-training, meaning that pretraining is resumed or restarted under a fairness constraint. This is the one option that the fine-tuning-failure result does *not* rule out; indeed it follows from that result, because if alignment bias is fixed in the representation then the representation has to be learned again. It is also why the Tier 2 methods of Section 4 intervene at the contrastive stage rather than afterwards. Re-training is expensive and out of reach for most clinical sites, and if it is run on the same skewed corpus without a constraint it simply restores the bias (17, 30). The second route is retrieval-augmented generation (RAG), now widely used to ground medical LVLMs on external evidence at inference without changing their weights. MMed-RAG, for example, reports large gains in factual accuracy across radiology, ophthalmology, and pathology (31). For hallucination this is useful, but for fairness RAG is a Tier 3 method with two limits. It does nothing to the Tier 2 representation or to the demographic information encoded there, and it opens a new bias surface at the retrieval step, since a knowledge base that over-represents majority groups will have that skew passed through by retrieval. RAG has in fact been shown to leave fairness unchanged, and sometimes to worsen it, even where accuracy improves (32). Neither route removes alignment-stage bias, which again points to pretraining as the place where the work has to be done.

### 2.3 Tier 3: inference-time bias

The first two tiers are structural. The third is behavioural: it appears only once a deployed model is placed in a conversation and subjected to the social and cognitive pressures that come with one.

**Sycophancy.** The most alarming finding in the recent literature is also the easiest to state. Asked to confirm a wrong answer by a confident user, models frequently do so, even when the image plainly says otherwise. EchoBench tested a spread of proprietary, open-source, and medical-specialist models, and found the behaviour everywhere (19). The best proprietary system, Claude 3.7 Sonnet, reversed a correct answer on 45.98% of adversarial trials, and GPT-4.1 on 59.15%. Medical-specialist models such as LLaVA-Med exceeded 95%. Some of the specialist failure is really a collapse in instruction-following rather than a clean reversal, but the clinical reading is the same: the models built specifically for medicine were the least able to hold their ground. Authority cues (a user presenting as a senior physician) were the most effective trigger, which is exactly the wrong property for a tool meant to support clinicians. In our view this is the most underrated patient-safety result in the field, and it is invisible to any accuracy- or parity-based evaluation.

**Cognitive-bias susceptibility.** BiasMedQA reworded United States Medical Licensing Examination (USMLE) questions to plant classic cognitive biases and tested several language models on them (20). False-consensus framing cost 24.9% accuracy on average, with recency, frequency, and status-quo framings close behind. The instructive detail is PMC-Llama 13B: the most heavily medical model in the set was the least accurate on the clean questions while only moderately susceptible to the framing manipulations. Medical knowledge and resistance to manipulation, in other words, are separate capabilities, and training that buys the first does not automatically buy the second.

**Demographic prompts and hallucination.** Inserting a patient’s demographic details into a prompt can shift a model’s diagnostic threshold unevenly across groups (6, 33), and models give less assertive advice to some demographic groups on otherwise identical cases (16). Hallucination compounds the problem: specialist LVLMs have been reported to fabricate more often than general models, and more still under misleading prompts (34). Because those fabrications need not be evenly distributed across groups, hallucination functions as an amplifier of the other Tier 3 effects.

### 2.4 Cross-tier mechanisms

The tiers mark where a bias enters the pipeline. They are not meant as sealed boxes, and a few mechanisms are generated at one stage but only cause harm at another. We noted one already: false-negative amplification is set up by Tier 1 note summarization but produced by the Tier 2 objective, and we assign it to Tier 2 because that is where the harm is generated. Dataset shift is the clearest case, and the one that matters most for deployment. When the demographic mix of the population a model is used on differs from that of its pretraining corpus, the cause is a Tier 1 property, an imbalance fixed in the training data, but the harm appears only at Tier 3, once the model meets a population it was not built for. An audit run on held-out data from the training distribution will not see it at all. What makes this case difficult is that the two obvious fixes work against each other. Yang and colleagues report that models leaning on demographic shortcuts can look fair on in-distribution test data and still lose both accuracy and fairness once they are applied to new populations, so that removing the shortcut to satisfy an in-distribution fairness metric can hurt real-world performance (35). For this reason we read the tier labels as the primary point of entry and the most practical place to intervene, not as exclusive categories, and we mark cross-tier cases wherever they come up. A taxonomy organized by locus is what brings these cases into view in the first place, because it forces the question of where a shared bias is best cut.

## 3 Evaluation frameworks

The benchmarks have multiplied quickly, and they do not agree. Harvard-FairVLMed was the first dataset built specifically for vision-language fairness: 10,000 fundus image-report pairs for glaucoma, labelled with four protected attributes (race, gender, ethnicity, and language), and it remains the standard testbed for Equity-Scaled Area Under the Curve (ES-AUC) (30). FMBench moved the target to open-ended generation, with 30,000 Visual Question Answering (VQA) pairs and 10,000 image-report pairs (7). Its useful contribution was to show that lexical metrics such as BLEU and METEOR can move in the opposite direction from clinically grounded quality judgements. The NIH 6×200 set, balanced by age and sex, was the vehicle for the Tier 2 fine-tuning result discussed above (17).

Two benchmarks push past parity. CARES is broader: roughly 41,000 question-answer pairs across 16 imaging modalities and several medical LVLMs, scored at once on truthfulness, fairness, safety, privacy, and robustness (36). Its most useful result is that these dimensions move together. A model that is unfair tends to be less robust and less factual too, so fairness cannot be bolted on after the fact. EchoBench, already discussed, measures sycophancy over 2,122 cases and 90 adversarial prompts (19). CALIN isolates demographic gaps in calibration, as distinct from accuracy, under few-shot in-context learning (37), and hallucination benchmarks such as MedVH probe fabrication under adversarial input (34).

The uncomfortable implication is that “fair” is underdetermined. The same model can pass Harvard-FairVLMed on parity, fail CALIN on calibration, carry a wide certainty gap on the cross-modal Maximum Mean Discrepancy benchmark (CMAC-MMD) (29), hallucinate on MedVH, and capitulate on EchoBench, all at once. Until the community settles on what to measure, gains on one axis will keep masking losses on another. This is the problem CONSORT-AI was created to resolve for clinical trials, and it calls for the same kind of fix.

## 4 Mitigation strategies

Mapped onto the taxonomy (Table 1), the mitigation work has an obvious blind spot: almost every method targets a single tier, and none covers all three.

At Tier 1 the goal is to fix the corpus. One study of structured metadata curation reports a result worth dwelling on: adding every available patient attribute indiscriminately can degrade fairness rather than improve it, so curation has to be selective and deliberate (38). Subgroup Distribution Aligned Tuning balances rare subgroups using a text-to-medical-image diffusion model, at the cost of inheriting whatever bias the generator carries (39). Med-UniC adds target-language vocabulary and a negative-free cross-lingual regularizer (27). None of this is sufficient on its own: leave the contrastive objective unconstrained and the bias returns even on balanced data (17, 30).

Tier 2 is where most of the recent ideas sit. FairCLIP equalizes subgroup distributions in the shared space with a Sinkhorn optimal-transport penalty (30). Robust FairCLIP adds Dynamic Bad Pair Mining, a loss-history filter for the noisy pairs created by note summarization, and reports up to 8.6% better ES-AUC (28). Fair-Mixture-of-Experts routes around biased visual patches with sparse experts and reportedly improves accuracy and fairness together on Harvard-FairVLMed (40). DualFairVL takes the lightweight route, using orthogonal text anchors and a cross-attention hypernetwork, and reports competitive fairness with only a few million trainable parameters (41). That small footprint is its main practical attraction, since most clinical sites cannot retrain a foundation model. CMAC-MMD attacks the certainty gap directly with a Maximum Mean Discrepancy penalty across intersectional subgroups, reporting roughly a 48% reduction in the skin-lesion missed-diagnosis gap without needing demographic labels at test time (29). Two further lines drop the requirement for demographic supervision entirely (42) or fold fairness regularization into LoRA (43).

Tier 3 methods have one clear advantage: they require no retraining and so apply to closed-source models. CALIN narrows calibration gaps by up to 90% through bi-level null-input probing, with no labels (37). VIPER strips social cues from the prompt and re-anchors the answer on the image; it helps, but only partly, and it is weakest against precisely the authority and mimicry cues that matter most in a clinic (44). Lighter touches recover some ground: few-shot bias education (20) and explicit fairness instructions (16). Integrated trust frameworks attempt detection, explanation, and mitigation together, though with thin validation so far (45). Retrieval-augmented generation belongs here too. It grounds outputs on external evidence and sharply reduces hallucination (31), but it leaves the Tier 2 representation untouched and is not fairness-neutral, because an unrepresentative knowledge base lets the retrieval step carry demographic skew through to the output (32). The common weakness is that these are symptomatic treatments: they leave the Tier 2 representation untouched.

## 5 Discussion and future directions

The most useful thing the taxonomy reveals is a structural gap rather than a missing algorithm. Every published *mitigation method* above intervenes at a single tier. This is a statement about the fragmentation of solutions, not a claim that each bias *mechanism* is confined to one stage; as Section 2.4 shows, some mechanisms span tiers. What the field lacks is a training regime that couples Tier 1 data balancing, Tier 2 alignment regularization, and Tier 3 robustness in a single objective. A reinforcement-learning formulation that penalizes demographic disparity and sycophancy together, scored against a multi-tier benchmark suite, is one plausible route and a realistic two-to-three-year target.

Several narrower priorities follow. Intersectional reporting should be the default rather than the exception. Most evaluations still vary one attribute at a time. The few that do not, CMAC-MMD among them (29), confirm the older clinical finding that compound disadvantage exceeds the sum of its parts (10). Stratification by age, sex, and race, with sensitivity analyses where the cells are thin, should be expected of any submission. Metrics need similar discipline. The literature now runs on at least seven fairness measures, among them ES-AUC, equality-of-odds difference, Demographic Parity Difference, Expected Calibration Error (ECE) difference, certainty gap, and sycophancy rate. A model can win on one and lose on another. A minimal common set of ES-AUC, ECE difference, and sycophancy rate, in the spirit of CONSORT-AI, would make results comparable; this is a coordination problem, solvable in a year or so, not a research problem.

The harder problems are not algorithmic at all. Med-UniC covers English and Spanish, but more than 6,000 languages have no presence in medical VLP, and the Global South contributes under 0.7% of the major datasets (24). No amount of loss-function engineering substitutes for the data-collection and computing investment those numbers imply; this is a five- to ten-year undertaking, and it aligns with the WHO’s recommendations on participation by low- and middle-income countries (8). The specialization result, finally, deserves to be read as a design warning. LLaVA-Med’s sycophancy far exceeds that of the general-purpose Claude 3.7 Sonnet (19), medical fine-tuning has been linked to higher hallucination rates (34), and BiasMedQA’s most specialized model was its least accurate on clean questions (20). The thread running through all three is that medical competence and epistemic robustness are not the same objective and may trade off against each other; instruction tuning that optimizes the first while ignoring the second is storing up trouble.

Regulation will force part of this regardless. The EU AI Act will require bias evaluation for high-risk medical AI from August 2026 (21), and the WHO guidance already provides an international reference point (8). The MIDRC catalogue of imaging biases (12) has not yet been adapted to the vision-language setting; doing so, and designing fairness evaluations that a regulator would accept, is work the field should begin now rather than after the deadline.

## 6 Conclusion

Medical VLMs need a fairness account built for their architecture, not borrowed from the unimodal past. The three-tier taxonomy offered here is meant as that account: it says where bias enters, which instruments detect it, and which remedies are appropriate at each stage. The findings that matter most are the uncomfortable ones. Mitigation is fragmented, and no method yet reaches across all three tiers. The bias that does the most damage is encoded during cross-modal pretraining and cannot be removed afterward, which makes the alignment interface the place to intervene. And the models trained hardest on medicine are not the ones that hold up best under pressure from a confident user; they may be the worst. Fairness in this setting is not a problem to be solved once but a property to be maintained, from the first data-collection decision to the last clinical interaction.
